# Successes and Challenges of Optimal Trauma Care for Rural Family Physicians in Kansas

**Published:** 2017-02-15

**Authors:** Gina M. Berg, Cheryl Dobson, Felecia A. Lee, Ashley M. Hervey, Rick Kellerman

**Affiliations:** 1 Department of Family and Community Medicine, University of Kansas School of Medicine-Wichita, Wichita, KS; 2 Department of Family and Community Medicine, Wesley Medical Center, Wichita, KS

**Keywords:** rural health, advanced trauma life support care, family physicians, patient transfer

## Abstract

**Introduction:**

Kansas has a regionalized trauma system with formal mechanisms for review, however, increased communication with rural providers can uncover opportunities for system process improvement. Therefore, this qualitative study explored perceptions of family medicine physicians staffing emergency departments (ED) in rural areas, specifically to determine what is going well and what areas needed improvement in relation to the trauma system.

**Methods:**

A focus group included Kansas rural family physicians recruited from a local symposium for family medicine physicians. Demographic information was collected via survey prior to the focus group session, which was audiotaped. Research team members read the transcription, identified themes, and grouped the findings into categories for analysis.

**Results:**

Seven rural family medicine physicians participated in the focus group. The majority were male (71%) with the mean age 46.71 years. All saw patients in the ED and had treated injuries due to agriculture, falls, and motor vehicle collisions. Participants identified successes in the adoption and enforcement of standardized processes, specifically through level IV trauma center certification and staff requirements for Advanced Trauma Life Support training. Communication breakdown during patient discharge and skill maintenance were the most prevalent challenges.

**Conclusions:**

Even with an established regionalized trauma system in the state of Kansas, there continues to be opportunities for improvement. The challenges acknowledged by focus group participants may not be identified through patient case reviews (if conducted), therefore tertiary centers should conduct system reviews with referring hospitals regularly to improve systemic concerns.

## Introduction

In 2015, traumatic injuries were the leading cause of death in the United States for children and adults aged 1 – 34 years.^1^ In the United States, trauma care operates within regionalized systems, which are managed on a state-by-state basis. Regionalization is based on the idea that trauma patients who present to rural or smaller hospital facilities can be triaged, stabilized, then transferred to a tertiary facility for definitive care.^2^ Within the regionalized systems, the American College of Surgeons (ACS) sets forth requirements for healthcare facilities to be classified as a level I, II, III, or IV trauma center.^3^ Criteria are the most rigorous for a level I facility, which is typically a large, high-volume urban hospital and there is a stepwise progression through level IV, which is typically a lower-volume rural hospital.

Nearly one-sixth of Americans live in a rural area, with 46.7 million Americans living greater than one hour away from the closest level I or II trauma center.^4^ Patient outcomes consistently have been shown to be worse when traumas occur in rural areas.^5^–^9^ Challenges include the availability, training, and skills-maintenance obstacles of the local emergency teams^10^ or long travel distances to larger trauma centers.^11^,^12^ Kansas is a state in which rural trauma is particularly important, as more than 94% of Kansas’ 105 counties are classified as either rural or frontier.^13^ In 2014, seventy percent of all Kansas traffic trauma fatalities occurred in a rural area,^14^ and approximately twenty-five Kansans require the care of a trauma center on any given day.^15^ In 1999, the Kansas legislature passed a law mandating the development and implementation of a regionalized state trauma system, consisting of six regions,^16^ with a state Advisory Committee on Trauma (ACT).^17^ As of 2016, there were three level I trauma centers (two located in Wichita, one located in Kansas City), two level II trauma centers, five level III trauma centers, and thirty-three level IV trauma centers.^18^

Level IV trauma centers are often Emergency Departments (ED) in small rural hospitals and frequently are staffed by family medicine physicians.^19^,^20^ A review of rural ED staffing in several states, including Kansas,^21^ noted a variety of ED staffing models, including traditional rotations of local medical staff, hiring of part-time or full-time ED physicians, use of mid-level practitioners or resident physicians, non-local locum tenens providers, or a combination of the aforementioned models.^21^–^25^ Moreover, rural EDs are less likely to utilize Emergency Medicine residency-trained/board-certified physicians than urban EDs.^23^,^26^,^27^ Rural emergency care in Kansas historically has been provided by family physicians.^22^

Kansas has a regionalized trauma system, with formal mechanisms for review and feedback on patient cases. Discussion outside of the formal review process, however, can uncover opportunities for system process improvement. Thus, this qualitative study explored perceptions of family medicine physicians staffing EDs in rural areas, specifically to determine what is going well and what areas still need improvement in relation to a trauma system.

## Methods

### Participants

Focus group participants were recruited from a local symposium for family medicine physicians in Kansas. Recruitment strategies included: 1) a letter to conference enrollees within two weeks prior to the symposium, and 2) inviting participants during the symposium announcements. Informed consent was obtained from each participant prior to beginning the focus group. A $20 gift card was given to each participant.

### Materials and Methods

The focus group session was conducted by a trained facilitator, lasted approximately one hour, audiotaped, and professionally transcribed with all identifying information removed. The focus group discussion included two queries (what is going well and what needs improvement) in the specific areas of: pre-hospital care, rural hospital care, transfers to tertiary care, skills maintenance, and concerns regarding special populations (pediatric and geriatric). Suggestions were requested for research and/or education. Demographics were assessed via a survey and included questions concerning skill maintenance and commonly treated mechanism of injuries.

### Data Analysis

Two independent reviewers analyzed focus group comments for qualitative data analysis. After individually reviewing transcripts, the reviewers discussed agreements and disagreements in success and challenge concepts and came to a consensus. Descriptive statistics were summarized using frequencies (percentages) and means (standard deviations). Statistical analyses were performed using SPSS for Windows, Version 20.0 (IBM, Armonk, NY).

## Results

Seven rural family medicine physicians participated in the focus group. Of the seven participants, the majority were male (5/7, 71%) with the mean age 46.71 years (SD = 16.62). The majority (6/7, 86%) were confident in their ability to manage major trauma. However, half (4/7, 57%) were concerned with skills maintenance due to infrequent exposure. All the physicians reported having treated patients in the ED with injuries due to agriculture, falls, and motor vehicle collision.

### Pre-Hospital Care

While discussing pre-hospital care, several participants expressed that advanced notification of incoming traumas, correct on-scene triage of patients, and air medical transport capability at the scene have led to improved trauma care. Several participants also mentioned they liked that the region has standardized triage notifications in place so that transport decisions automatically are made at the scene regarding whether transport will be made to the closest rural hospital or directly to a center with a higher trauma designation. The pre-hospital notification system of text messages implemented as part of attaining level IV trauma center certification resulted in improved delivery of trauma care for one participant’s facility. Partnership with the Kansas Board of Emergency Medical Service (EMS) was reported as a positive factor, as this has resulted in assistance with emergency services protocol and process enhancement for many facilities.

Participants discussed areas within pre-hospital care that presented challenges, including lack of protocol consistency and training among EMS providers, as well as availability of EMS resources (EMTs/paramedics and ambulances). Frustration was expressed with lack of adequate pre-hospital notification when there is an incoming trauma, delayed notification about ambulance or staff availability, and the possibility of their facility being passed over inappropriately in favor of a larger facility. Several participants mentioned that many first-responders are volunteers, therefore, the level of training and familiarity with protocols vary.

### Rural Hospital Care

Many commented on successes including improvements resulting from achieving level IV trauma center certification, specifically, quarterly inter-disciplinary meetings for case review and process improvement recommendations. Additional comments noted resource-focused communication (unavailability of key elements for trauma care, such as radiology or lab), efficient team assembly (text notification of an incoming trauma), correct pre-hospital triage (patients appropriately matched to the level of care that their facility is equipped to provide), and the use of telemedicine.

Challenges identified at rural hospitals included the need for appropriately skilled providers, administrative reluctance to put a hospital on diversion (when necessary), and skill maintenance.

### Tertiary Care Transfer

As participants discussed transfers to tertiary care centers for definitive care, several participants felt well-supported by the larger hospitals and overall have good communication with the providers. Specifically, they mentioned consulting with tertiary hospital providers, even when transfer was not planned. A reported success was the utility of the ‘one call system’ for contacting level I trauma centers in the region, saying it is helpful when preparing a patient for transfer. Participants also appreciated follow-up on the care of their patients, such as daily reports or written documentation of discharge. Benefits of having specialty physicians visit and hold clinics at rural hospitals were reported, stating it helps the referring physician build a rapport and feel more comfortable when transferring patients to his/her care. The specialty physicians also provide significant help with post-discharge follow-up after a patient has been treated at a tertiary care center.

Discussion regarding challenges identified in transfers was predominantly centered on concerns surrounding communication with the tertiary trauma center. Participants expressed frustration over difficulty obtaining discharge summaries in a timely manner. Further, the length and structure of the summary were noted as being cumbersome for referring physicians to locate information easily such as discharge diagnosis and new medications. Rural providers’ frustrations are compounded when the physician feels that he/she made an effort to provide the receiving facility with the patient’s records in a timely manner but may not hear anything until the patient returns home for follow-up care. This lack of information causes frustration for the provider and patient. It was mentioned that sometimes tertiary care centers have case managers who keep the referring physician in the loop and help with communication, but this is not always the case.

Another challenge complicating patient transfers is strained relationships between the sending and receiving physicians. Several focus group members expressed they have been criticized or have perceived the receiving physician was being judgmental of the care provided in the rural setting or the decision to transfer the patient. Participants stated there can be a perception that they are *“dumping”* their patient on receiving facilities, but they are trying to transport while stable. Sometimes the referring physician decides it is necessary to transfer a stable patient because he/she wants to get the patient to the definitive care facility (potentially several hours away) in case the patients’ condition deteriorates. It was observed that this is likely due to a lack of understanding of the resources available to care for the trauma patient in the rural setting. Another patient transfer issue related to the patients’ reluctance to leave their home community to go to a larger city for medical care. This can present challenges for the rural trauma provider who is trying to coordinate appropriate care of the patient.

### Skill Maintenance/Continued Education

Reported successes in this area included facility-specific practices, such as conducting mass casualty drills, as well as having trauma surgeons from level I trauma centers in the state travel to their rural hospital to conduct training and review protocols with the rural healthcare team. Several participants reported that skills maintenance was a major challenge for them. One participant commented that it was helpful to have trauma surgeons come out once a year to go through rarely used procedures. Others mentioned that continued education was addressed in their institutions by implementing requirements for staff members/providers to participate regularly in advanced trainings such as Advanced Trauma Life Support (ATLS), Advanced Cardiac Life Support (ACLS), and Pediatric Advanced Life Support (PALS). Other participants cited attending and networking at meetings, webinars, and conferences as being helpful resources for continued education. Additionally, several participants expressed concern about having the opportunity to participate in the care of enough trauma patients to maintain their skills. Others voiced uncertainty as to which specific trauma training, ATLS or American Board of Emergency Medicine (ABEM) training/certification, is most appropriate for them as family medicine physicians who are providing trauma care in a rural setting.

### Special Populations (Pediatric and Geriatric)

During the focus group, participants were asked specifically about special trauma populations, including pediatric and geriatric patients. When discussing pediatric trauma patients, several participants voiced their lack of confidence with regards to skills in handling these patients, due to their rare occurrence. One participant put it simply, *“They are terrifying.”* Another concern was consulting with specialists outside of the physician’s trauma system. They are communicating with new providers that they do not have a relationship with and this can lead to a breakdown in communication.

Geriatric trauma patients present unique challenges; *“They won’t stop falling.”*

The decision to transfer a geriatric trauma patient to a tertiary facility for advanced care can be complicated by end-of-life issues. A patient’s wishes regarding end-of-life care can be dynamic and must be revisited with each hospital visit and carefully documented.

## Discussion

The purpose of this focus group was to explore perceptions of family medicine physicians staffing EDs in rural areas to determine what is going well and what areas still need improvement in relation to a trauma system.

In the area of pre-hospital care, improved triage protocols, timely notification of incoming trauma patients, and availability of air-ambulance transport from the scene for critical patients contributed significantly to improved rural trauma care delivery. However, many providers experience significant pre-hospital care challenges, particularly related to EMS provider level of training and availability of ambulances and first-responders. These challenges, including providing quality pre-hospital trauma care in a rural setting, are well-documented in the literature. Many EMS providers are volunteers with full-time jobs and families, and the rural EMS provider may not have ample exposure to trauma patients for adequate skills maintenance and familiarity with protocols.^5^,^11^,^28^ Potential solutions to these challenges include making additional human resources available to dispatch (e.g., law enforcement and fire department personnel). In addition, providing rural personnel with engaged medical oversight to answer questions and help hone skills can effectively maintain skill performance.^11^

With regards to what happens within the rural hospital, systematic improvements were reported widely among participants whose facilities had undergone standardization criteria for level IV certification.^3^ Additional positive factors were continuing education (interdisciplinary patient case reviews) and improved communication (both resource availability and efficient team assembly). While the use of telemedicine was discussed only briefly during the focus group, it has been explored in rural trauma literature. The expansion of telemedicine initially was expected to occur rapidly, however, challenges related to funding and state licensing are attributed to its lackluster growth.^29^,^30^ The use of telemedicine in rural trauma care holds great potential and may be used in the future to aid rural hospitals in providing optimal evaluation, treatment, and transfer of trauma patients.^31^

Issues surrounding transferring patients to a tertiary facility provoked the most discussion among focus group participants, and experiences varied. Good communication with providers at tertiary care facilities and specialists traveling to rural hospitals were cited as positives, as was the ‘one call system’ for contacting level I trauma centers in the region. Areas identified for improvement included the timely receipt of concise discharge instructions, updates while the patient is hospitalized, and communication (free of criticism or judgment) between the sending and receiving physicians. Rae et al.^32^ described a highly successful system used by Harborview Medical Center in Seattle, Washington, to provide timely patient information to referring hospitals. The system, called U-link, is managed by the level I trauma center and designed to allow authorized individuals from the referring hospital to have HIPAA-compliant access to a trauma patient’s records for the purpose of improved post-discharge follow-up and provider education. Implementation of a similar system locally would require investigation regarding cost and risk to patient privacy, but could provide substantial benefit to referring physicians.

Skills maintenance/continued education topics identified as being positive included required ATLS/ACLS/PALS education for staff, as well as trauma surgeons traveling from tertiary care centers to rural facilities to train trauma teams. McCrum et al.^33^ observed that while adherence to ATLS guidelines in trauma care is tied to improved patient outcomes, the reality is that often ATLS protocol is not followed in the rural trauma setting for a variety of reasons, including lack of availability of ATLS training and infrequency with which rural providers are able to practice trauma skills. Some hospitals represented by the focus group are addressing this issue, as participants reported all staff are now required to be ATLS certified. However, trauma volume at rural hospitals is often insufficient for skills maintenance which remains a concern. The use of simulation modules provides rural personnel with opportunities to maintain skills.^34^ In addition, focused and directed educational programs that emphasize skill practice can improve skill performance.^35^

Participants discussed issues pertaining to the care of special trauma populations. Pediatric patient concerns included lack of comfort in caring for critically ill children, inadequate exposure to pediatric trauma patients for skills maintenance, and the need to work with specialists with whom the physician may not be familiar. Limited exposure to pediatric patients is a common barrier to ED providers staying current on knowledge, as the majority does not have access to pediatric-specific emergency training. Curran et al.^36^ recommended the use of a web-based knowledge exchange focused on pediatric emergency care to help rural providers access current research and increase communication with other providers of pediatric emergency care. Communication is particularly important in pediatric trauma care, specifically between the rural facility and tertiary care facility. Chwals et al.^37^ noted approximately 91% of computerized tomography (CT) scans performed at a rural hospital are repeated once the patient reaches the larger definitive care facility, subjecting the young patient to increased radiation.

Issues raised by participants with regards to geriatric trauma patients included the need for end-of-life discussions and the frequency with which elderly patients experience falls. The literature reveals the complexity of trauma care related to the geriatric population, including challenges with adequate assessment of severity of injuries (particularly head injuries),^38^ management of age-related co-morbidities, and corresponding timing of transfer to a tertiary care facility.^39^ The subject of trauma and the elderly will gain attention in the coming years, as the US Centers for Disease Control and Prevention predicts there will be twice as many elderly people (aged 65 and older) alive in the year 2050 as there were in 2010.^40^

### Limitations

The results of this focus group discussion may not represent all rural hospitals providing emergency and trauma care due to the small number of participants (seven), the recruitment strategy (family medicine physicians attending local symposium), and all physicians providing care in Kansas. Due to the diverse background of rural providers, praises or concerns mentioned by an individual participant may not be shared by other providers.

## Conclusion

Even though regionalized trauma centers are established in the state of Kansas with quality review and process improvement in place, there continues to be opportunities for improvement within the system. Positives were adoption and enforcement of standardized processes through level IV trauma center certification and staff requirements for ATLS training. Communication breakdown (in terms of patient discharge information) and skill maintenance were the most prevalent challenges noted. These issues specifically may not be identified through inter-facility patient case reviews (if conducted), thus tertiary centers should conduct system reviews with referring hospitals regularly to address regional systemic concerns.
